# Mechanical, Electrical and Magnetic Properties of Ferrogels with Embedded Iron Oxide Nanoparticles Obtained by Laser Target Evaporation: Focus on Multifunctional Biosensor Applications

**DOI:** 10.3390/s18030872

**Published:** 2018-03-15

**Authors:** Felix A. Blyakhman, Nikita A. Buznikov, Tatyana F. Sklyar, Alexander P. Safronov, Elizaveta V. Golubeva, Andrey V. Svalov, Sergey Yu. Sokolov, Grigory Yu. Melnikov, Iñaki Orue, Galina V. Kurlyandskaya

**Affiliations:** 1 Ural State Medical University, Yekaterinburg 620028, Russia; Feliks.Blyakhman@urfu.ru (F.A.B.); t.f.shkliar@urfu.ru (T.F.S.); sergey.sokolov@urfu.ru (S.Y.S.); 2 Institute of Natural Sciences and Mathematics Ural Federal University, Yekaterinburg 620002, Russia; safronov@iep.uran.ru (A.P.S.); golubeva.elizaveta.v@gmail.com (E.V.G.); andrey.svalov@urfu.ru (A.V.S.); grisha2207@list.ru (G.Y.M.); 3 Scientific and Research Institute of Natural Gases and Gas Technologies—Gazprom VNIIGAZ, Razvilka Leninsky District, Moscow Region 142717, Russia; n_buznikov@mail.ru; 4 Institute of Electrophysics, Ural Division RAS, Yekaterinburg 620016, Russia; 5 Advanced Research Facilities (SGIKER), Universidad del País Vasco UPV-EHU, 48080 Bilbao, Spain; inaki.orue@ehu.eus; 6 Departamento de Electricidad y Electrónica and BCMaterials, Universidad del País Vasco UPV/EHU, 48080 Bilbao, Spain

**Keywords:** magnetic nanoparticles, magnetic biosensors, ferrogels, magnetic multilayers, giant magnetoimpedance

## Abstract

Hydrogels are biomimetic materials widely used in the area of biomedical engineering and biosensing. Ferrogels (FG) are magnetic composites capable of functioning as magnetic field sensitive transformers and field assisted drug deliverers. FG can be prepared by incorporating magnetic nanoparticles (MNPs) into chemically crosslinked hydrogels. The properties of biomimetic ferrogels for multifunctional biosensor applications can be set up by synthesis. The properties of these biomimetic ferrogels can be thoroughly controlled in a physical experiment environment which is much less demanding than biotests. Two series of ferrogels (soft and dense) based on polyacrylamide (PAAm) with different chemical network densities were synthesized by free-radical polymerization in aqueous solution with *N*,*N*’-methylene-diacrylamide as a cross-linker and maghemite Fe_2_O_3_ MNPs fabricated by laser target evaporation as a filler. Their mechanical, electrical and magnetic properties were comparatively analyzed. We developed a giant magnetoimpedance (MI) sensor prototype with multilayered FeNi-based sensitive elements deposited onto glass or polymer substrates adapted for FG studies. The MI measurements in the initial state and in the presence of FG with different concentrations of MNPs at a frequency range of 1–300 MHz allowed a precise characterization of the stray fields of the MNPs present in the FG. We proposed an electrodynamic model to describe the MI in multilayered film with a FG layer based on the solution of linearized Maxwell equations for the electromagnetic fields coupled with the Landau-Lifshitz equation for the magnetization dynamics.

## 1. Introduction

Magnetic biosensors are compact analytical devices incorporating a biological or biologically derived sensitive element integrated in or associated with a physicochemical transducer employing magnetic materials and magnetic fields [1,2]. These devices are well suited to the requirements of biomedical applications where there is an increasing number of different tests and demand for simple measurement protocols [3]. They are not an alternative to complex medical equipment but in many cases they are cheap and well adapted to society’s need to make medical care promptly available to everyone. The first type of magnetic transducer was introduced for magnetic permeability measurements in bioanalysis by inductance measurements with a Maxwell bridge [4]. Nowadays, different magnetic effects have been shown to be capable of creating magnetic biosensors: anisotropic magnetoresistance, giant magnetoresistance, tunneling magnetoresistance, spin-valves, magnetoelastic effect, inductive effect, the Hall effect and magnetoimpedance (MI) [5,6,7,8,9,10,11,12]. 

Taking the simplest classification (in accordance with the working principle) magnetic biosensors can be divided into two groups: biosensors based on magnetic labels and label-free detecting systems. The functional basis of the magnetic label detection is simple—the fringe fields of magnetic markers employed as biomolecular labels provide a means for the transfer of biological information. In the case of biomedical applications, superparamagnetic nanoparticles (MNPs) must be provided in the form of water-based ferrofluids or ferrogels [13,14,15]. Apart from the classic configuration of magnetic bio-detection, in which all magnetic labels are placed at a certain distance from the surface of the sensitive element as a result of molecular recognition events, more complex configurations have also been tested. MNPs can be detected inside a living cell after intracellular uptake [11,16]. However, the detection of the MNPs incorporated into living tissues, has not yet been properly addressed. 

The principle reason for this delay is the sensitivity of the existing magnetic biosensors to a magnetic field. In this respect, giant MI [17,18] have attracted special attention as this phenomenon provides a basis for biosensors capable of detecting picotesla magnetic fields [19]. A superparamagnetic label (a uniformly magnetized sphere) can be roughly viewed as a magnetic dipole placed in the centre of a sphere [20]. The stray field generated by it is proportional to the |X|^−3^, where X is a distance between the dipole centre and the point under consideration. In the simplest magnetic biosensor, it is the distance between the centre of the nanoparticle and the surface of the sensitive element. This means that stray field value very rapidly decreases with an increase in the distance.

In the case of MNPs incorporated into living tissues, the evaluation of their stray fields becomes a very complex problem as the MNPs are situated at different distances and the majority of them are far from the surface of the sensitive element. In living cells after intracellular uptake of MNPs, this distance becomes in the order of at least 50 μm [21]. The detection of the MNPs incorporated into living tissues should take into consideration distances of at least 1 mm order. Analysis of long-range and short-range adhesive interactions of the streptavidin-biotin bond (a typical example of the molecular recognition event widely employed in biosensing) gives energy of about 31 kT, and its effective length is approximately 9 Å [22]. This means that for the development of specified biosensors for the detection of MNPs incorporated into living tissues we must make a step from about 1 nm to 1 mm distances keeping in mind |X|^−3^ law for the stray field strengths. This is why the sensitivity of magnetic biosensors with respect to the applied field becomes a crucial condition of successful label detection. 

The second problem for the detection of stray fields of magnetic particles incorporated into living systems is a very high contribution of the water signal to the dielectric properties of the tissue itself [21]. This means that the magnetic signal detection by the dynamic measuring technique requires extraction of a rather small signal from a significant background. 

As cells or living tissues are basically soft gels [23], the mechanical properties are important as well because deformations can be caused by the application of an external magnetic field in the case of FG. It might cause changes in interparticle distances and their configuration, which can change their dipolar interactions and magnetic response, therefore complicating the stray field interpretation.

Finally, biological samples such as cells and tissues have a number of restrictions for testing by physical methods. Living matter is a cluster of interconnected complex processes that is hard to consider separately. In order to solve this problem, mathematical and physical models are widely used in biophysical research. This strategy allows us to reproduce the selected features and properties of the living system important for each particular case. In particular, for the structural organization of cells and tissues, polyelectrolyte synthetic hydrogels can be used as a physical model [24,25]. The development of a new generation of magnetic biosensors is conditioned by the development of reliable samples mimicking some properties of the living systems. Ferrogels offer the possibility of following selected morphologies, while avoiding a huge variety of morphologies usually present in biological tissues, especially the tumor tissues affected by angiogenesis [26]. 

Recently we proposed to substitute biological samples at the first stage of development of the MI biosensor by a model material—synthetic hydrogel with a certain amount of MNPs. Although, successful detection and stray field evaluation has been possible in the case of thin film structures deposited onto rigid substrates [2,27], the experiments with large FG pieces confirmed the urgent need to develop a model for the description of the MI in multilayered films with a top FG layer. 

In the present work, we describe our experience synthesizing and characterizing maghemite Fe_2_O_3_ MNPs obtained by laser target evaporation technique and ferrogels incorporating MNPs in chemically crosslinked hydrogels. The deformation of ferrogels and their electrical and magnetic properties were comparatively analyzed. FeNi/Ti and FeNi/Cu multilayered sensitive elements were deposited onto rigid or flexible substrates and magnetoimpedance sensor prototype responses were measured with and without a top FG layer. An electrodynamic model of the MI in multilayered film with a FG layer based on the solution of linearized Maxwell equations for the electromagnetic fields coupled with the Landau-Lifshitz equation for the magnetization dynamics was proposed.

## 2. Experimental Method

### 2.1. Synthesis of Iron Oxide MNPs and Preparation of Ferrofluid

Iron oxide MNPs were synthesized by laser target evaporation method (LTE) using commercial magnetite (Fe_3_O_4_) (Alfa Aesar, Ward Hill, MA, USA) as a precursor. Magnetite powder with specific surface area 6.9 m^2^/g (average particle diameter 0.2 μm) was pressed in a pellet of 65 mm in diameter and 20 mm in height, which was then evaporated by a laser beam using a laboratory installation designed at the Institute of Electrophysics UD RAS (Yekaterinburg, Russian Federation). A Ytterbium laser with 1.07 µm wavelength operated in a pulsed regime with pulse frequency 4.85 KHz and pulse duration 60 μs was used for the evaporation. The average output power of irradiation was 262 W. The condensation of MNPs from vapor took place in a permanent flow of argon (5 L/min). The details of the synthetic method are elaborated elsewhere [28,29]. 

An electrostatically stabilized ferrofluid based on iron oxide MNPs was prepared in 5 mM of sodium citrate solution in distilled water. Suspension in an initial concentration 5 wt % was de-aggregated by ultrasound with permanent cooling using Cole-Parmer CPX-750 processor (Cole-Parmer Instruments Corp., Vernon Hills, IL, USA) operated at 300 W power output for 30 min. The average hydrodynamic diameter of aggregates in suspension was monitored by dynamic light scattering. After that, the suspension was centrifuged at 10,000 rpm for 5 min using Hermle Z383 centrifuge (Hermle-labortechnik, Wehingen, Germany) to remove remaining aggregates. The concentration of ferrofluid after centrifuging was 3.2 wt % of MNPs. This stock ferrofluid was then diluted with 5 mM sodium citrate solution to obtain different concentrations for the preparation of ferrogels with varying MNP content. 

### 2.2. Synthesis of Ferrogels

Ferrogels based on the polyacrylamide (PAAm) chemical network were synthesized by free-radical polymerization in an aqueous solution with *N*,*N*’-methylene-diacrylamide (Merck Schuchardt, Hohenbrunn, Germany) as a cross-linker. Two series of ferrogels (soft and dense) with different densities were obtained. The soft series was synthesized in 1.6 M solution of acrylamide (AAm) (AppliChem, Darmstadt, Germany) and cross-linker to monomer molar ratio was set at 1:100. In the dense series the concentration of AAm was 2.7 M and cross-linker to monomer molar ratio was 1:50. Further on these series are denoted as FG-I (soft) and FG-II (dense). In each series the monomer and the cross-linker were dissolved in several ferrofluids with a diminishing concentration of MNPs to obtain ferrogels with varying content of magnetic particles. Ammonium persulfate in 3 mM concentration was used as an initiator and *N*,*N*,*N*’,*N*’-tetramethylethylene diamine (TEMED) (SigmaAldrich Inc., St. Louis, MO, USA) in 6 mM concentration as a catalyst. Polymerization was performed at room temperature for 1 h in polyethylene probe tubes. After the synthesis, the ferrogels were taken out from the tubes and extensively washed in distilled water for two weeks with water renewal every two days until constant weight of the gel samples was achieved.

As the ferrogel samples swelled in the consequent washing cycles after the synthesis, the final concentration of MNPs in ferrogels changed with respect to the initial concentration set up in the synthesis. The concentration of MNPs in ferrogels was determined by the gravimetric analysis. First, the equilibrium swelling ratio of the ferrogels after their equilibration was determined. The weight, m_0_ of a swollen piece of gel approximately 0.5 g was measured using an analytical balance (Mettler-Toledo MS104S). Then, it was dried in an oven at 90 °C down to the constant weight of the dry residue *m*_1_. The apparent swelling ratio (*α*) of the ferrogel was calculated according to the equation:

(1) $$\alpha =\frac{m_{0}-m_{1}}{m_{1}}$$

The value of the swelling ratio was used for the calculation of the particle content in the swollen gel applying the equation:

(2) $$\omega =\frac{\gamma }{1+\alpha }$$

where *γ* stands for the weight fraction of particles in the dry residue, which was measured by a thermogravimetry analysis (TGA). 

The values of *γ* were also used for the calculation of the swelling ratio of gel matrix of ferrogels (*α*′) which was related solely to the PAAm network excluding solid MNPs:

(3) $$\alpha^{\prime }=\frac{\alpha }{\gamma }$$

The values of the swelling ratio of ferrogels and the weight fraction of magnetic particles in the swollen gel are given in Table 1. 

**Figure 1:**
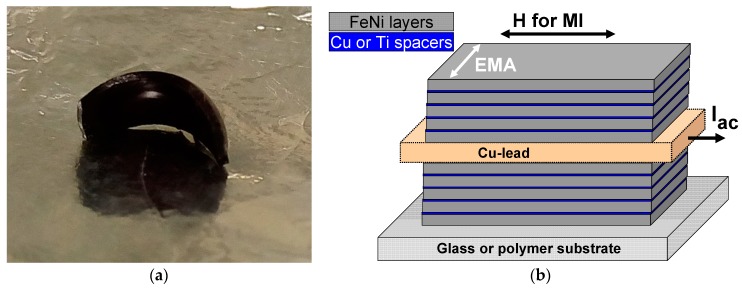
General view of FG-I-1 ferrogel 9 mm × 2 mm × 1 mm piece after 40 min drying in ambient conditions (**a**); Schematic description of the magnetoimpedance (MI) multilayered structures: EMA is the easy magnetization axis direction, close to the direction of a magnetic field applied during multilayered structure deposition; H is the external magnetic field direction for MI measurements, configuration of longitudinal magnetoimpedance (**b**).

The FG samples were cut in pieces of different shapes for different studies. Figure 1 shows a general view of FG-I-1 ferrogel sample after 40 min drying in ambient conditions. The geometry 10 mm × 2 mm × 1 mm was used for MI measurements with multilayered sensitive elements in order to test the magnetic biosensor prototype. 

### 2.3. Magnetic Multilayers Deposition

The magnetic MI samples were Fe_19_Ni_81_/Ti or Fe_19_Ni_81_/Cu multilayers, with a central non-magnetic copper layer deposited by dc-magnetron sputtering onto glass or polymer substrates. The optimum deposition conditions (both background pressure and a working Ar pressure) were previously discussed and proven [30,31]. A background pressure of 3 × 10^−7^ mbar and a working Ar pressure of 3.8 × 10^−3^ mbar were used in the present studies: [FeNi/Ti(6 nm)]_x_ or [FeNi/Cu(3 nm)]_x_ structures at the top and the bottom of the MI sandwich [32]. A transverse magnetic anisotropy was induced during the deposition process by the application of an in-plane constant magnetic field of 250 Oe for [FeNi/Ti(6 nm)]_x_ or 100 Oe [FeNi/Cu(3 nm)]_x_ multilayers. The shape of the MI sensitive elements was defined by the deposition conditions through the utilization of magnetic masks: 10 mm long and 0.5 mm wide rectangles. The long sides of the samples were oriented in a direction perpendicular to the direction of the applied magnetic field. Therefore, the induced magnetic anisotropy axis was formed parallel to the short side of the stripe in all of the samples. Figure 1b gives a description of the studied multilayers. As substrates we used either rigid (Corning glass) or flexible cycloolefin (ethylene-norbornene copolymer) polymer or COP, widely accepted for magnetic biosensors with microfluidic systems [30].

### 2.4. Methods

The specific surface area (Ssp) of powdered materials was measured by the low-temperature sorption of nitrogen (Brunauer-Emmett-Teller physical adsorption (BET)) using Micromeritics TriStar3000 analyzer (Micromeritics Instrument Corp., Norcross, GA, USA). Transmission electron microscopy (TEM) was performed using a JEOL JEM2100 microscope (JEOL Ltd., Tokyo, Japan) operated at 200 kV. The particles were spread on carbon-coated copper grids. The X-ray diffraction (XRD) studies were performed by Bruker DISCOVER D8 (Bruker, Billerica, MA, USA) diffractometer operated at 40 kV and 40 mA using Cu-Kα radiation (λ = 1.5418 Å), a graphite monochromator and a scintillation detector. The MNPs were mounted on a zero-background silicon wafer placed in a sample holder. A fixed divergence and anti-scattering slit were used. Bruker software TOPAS-3 with Rietveld full-profile refinement was employed for the quantitative analysis of all the diffractograms. The hydrodynamic diameter of MNPs/aggregates in suspension, the parameters of lognormal distribution, and zeta-potential were measured by the dynamic and electrophoretic light scattering using a particle size analyzer Brookhaven Zeta Plus (Brookhaven Instruments Corp., Holtville, NY, USA). TGA of dried ferrogels was performed using NETZSCH STA 409 thermal analyzer (NETZSCH Geratebau, Selb/Bavaria, Germany) by heating from 40 to 1000 °C at 10 K/min in an air flow of 20 mL/min.

The testing of mechanical characteristics of ferrogels in static and dynamic mode was performed using the laboratory setup. It comprised an electromagnetic linear motor for applying mechanical deformations, a semiconductor force transducer, and a semiconductor optical transducer for the measurement of the length of the ferrogel sample. A cylindrical ferrogel sample (~7 mm in length and ~90 mm^2^ in cross section) placed in a bath filled with distilled water was clamped vertically between two parallel plates connected to the levers of the force transducer and the linear motor. The measurement in a quasi-static mode was performed by a step-wise application of compressive deformation to a sample with force equilibration for at least 10 s at each step. The deformation step was 1–2% of the sample height and the total deformation of the sample was up to 15%. 

The testing of the electrical properties of ferrogels was performed using a laboratory installation for the electrical potential measurement in biophysical systems described elsewhere [33,34]. The measurement of the electrical potential of ferrogel was done by two identical Ag/AgCl tapered glass microelectrodes (~1 micron in tip diameter) typically used in biophysical studies for intracellular voltage measurement. The electrodes were single-pulled using a standard electrode puller, ME-3 (EMIB Ltd., Moscow, Russia) from thin-walled, single-barrel borosilicate capillary tubes, TW150F-6 (World Precision Instruments, Sarasota, FL, USA). The pulled electrodes were immersed in a 3 M KCl solution with the tip facing upward, so that the solution climbed to the tip by capillary action. One electrode was pinned into the ferrogel sample and the other was placed into outside water. The potential difference between microelectrodes was measured using an instrumental amplifier on the base of an integrated circuit, INA 129 (Burr-Brown, Dallas, TX, USA). To reduce the influence of electromagnetic interference on the potential difference measurement, special wire shields were provided around the measuring unit.

The in-plane magnetic hysteresis loops of the magnetic multilayers were measured by magneto-optical Kerr microscopy (MOKE device of laboratory design) for the whole sample size: 10 mm × 0.5 mm. The magnetization curves of ferrogels were measured by vibrating sample magnetometer (VSM: Faraday magnetometer of laboratory design) for samples of about 100 mg weight placed into a polycarbonate capsule. The capsule contribution was measured separately and carefully extracted from the M(H) data. All measurements were made at room temperature. 

**Figure 2:**
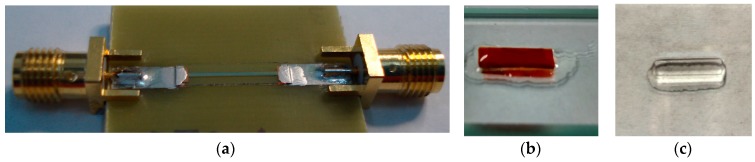
General view of the ribbon-based MI sensitive element installed into the “microstripe” line (**a**); general view of ferrogel (**b**) and gel; (**c**) pieces cut for MI measurements.

A thin film multilayered sensitive element was incorporated into a “microstripe” line using silver paint (Figure 2a). Figure 2b shows a general view of the ferrogel piece cut for MI measurements. A uniform external magnetic field of up to 100 Oe was created by a pair of Helmholtz coils and applied along the MI sensitive element elongated stripe, i.e., all measurements were made in longitudinal MI configuration (alternating current flowing parallel to the external magnetic field). The MI changes were calculated from the S_11_ reflection coefficient measured by a network analyzer (Agilent E8358A). An output power of 0 dB, corresponding to the amplitude of the excitation current across the sample of about 1 mA was used in all MI measurements. 

The calibration and mathematical subtraction of the test fixture contributions were carefully done following a previously established protocol [30]. Total impedance (Z) was measured as a function of the external magnetic field in a frequency range 0.1–300 MHz. MI ratio (4a) and MI ratio sensitivity were calculated as follows:

(4) $$\frac{\Delta Z}{Z}=100\times \frac{Z(H)-Z(H_{\max})}{Z(H_{\max})}$$

(5) $$S\left(\frac{\Delta Z}{Z}\right)=\frac{\delta (\Delta Z/Z)}{\delta H}$$

where *H*_max_ = 100 Oe and *δ* (Δ*Z*/*Z*) is the change in the total impedance MI ratio (*H*) = 0.1 Oe. For MI frequency dependence analysis, the changes of the maximum value of the total impedance Δ(*Ζ*/*Ζ*)_max_ was provided. The experimental error in the impedance determination was within 1%. Direct current resistivity (R_DC_) was also measured in all cases.

In our previous work, for MI measurements with ferrogels we used gel pieces of particular geometry of 1.0 to 0.5 g weight [2,35]. The measurements were strongly conditioned by the time as the gel/ferrogels changed mass rapidly, shrunk and lost water (see Figure 1a). The FG synthesized for the present study were kept in distilled water for storage. In the present work, we therefore developed a special protocol for MI measurements with gels and FG taking into account the mass loss of the piece of gel or FG of the same shape. The length was slightly shorter as about 0.1 mm from each end of the MI element was used for electrical connection. A special protocol was elaborated for MI measurements in order to minimize the experimental errors and ensure a reproducible procedure. First, the samples were cut in rectangles of 9 mm × 2 mm × 1 mm. Then each sample was taken out of the water and kept under ambient conditions for about 60 min, while its weight loss was measured by an analytical balance. The total time of the MI measurements in a decreasing field was limited to 3 min and characterized by a linear total mass loss of 10% with respect to the initial mass.

First, the MI responses were studied by measuring the multilayered sensitive element itself, i.e., without gel or ferrogel on top of it. Then, FG stripes (Figure 2b) were placed in the centre of the MI element one by one for different concentrations. Measurements with the gel were used for subtracting the corresponding signal from the ferrogel samples in order to evaluate the average contribution of the stray fields of magnetic particles in the spatial distribution corresponding to each ferrogel case. 

For the modeling of the MI response in a multilayered element in the presence of a ferrogel we proposed an electrodynamic approach in order to describe the MI in a multilayered thin film structure with a layer of ferrogel on top of it. This is why very thin FG samples of a small volume were used for the MI measurements—for larger samples, calculations were limited by the available computing capacity. The theoretical approach is based on the solution of linearized Maxwell equations for the electromagnetic fields coupled with the Landau-Lifshitz equation for the magnetization dynamics [18]. The influence of the ferrogel on the permeability of the multilayered element in the configuration of the MI sandwich is described in terms of an effective stray field created by MNPs.

## 3. Results and Discussion

### 3.1. Structural Characterization of Nanoparticles and Ferrogels

**Figure 3:**
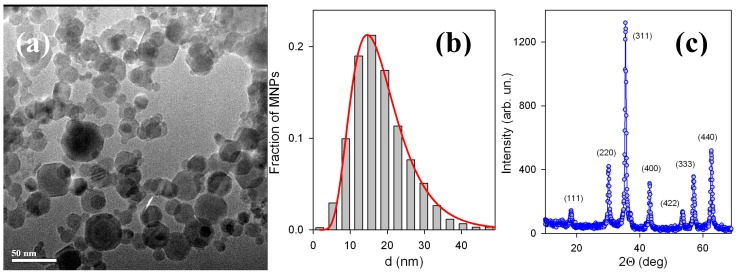
TEM image of iron oxide MNPs embedded in ferrogels (**a**). Particle size distribution obtained by the graphical analysis of 2160 particles (**b**); Inset (**c**) XRD pattern for MNPs with corresponding Miller indexes.

Figure 3a gives TEM image of synthesized MNPs and Figure 2b gives particle size distribution (PSD) histogram which was obtained by the graphical evaluation of the diameter of 2160 particles. The particles are spherical and non-coalescent. Only a few of the particles appeared to be hexagonal or to have hexagonal corners. PSD is well fitted by the following lognormal distribution function:

(6) $$PSD=\frac{3.38}{d}\exp\left[0.5{\left(\frac{\ln(d/17.3)}{0.413}\right)}^{2}\right]$$

The specific surface area of MNPs was 64 m^2^/g. The surface average diameter of MNPs, calculated from this value using the equation d_s_ = 6/(ρ × S_sp_) (ρ = 4.6 g/cm^3^ being iron oxide density) was 20.7 nm. This was in fair agreement with the value d_s_ = 24.4 nm, obtained using PSD (Equation (5)). The discrepancy between these two values apparently stems from the deviation from the spherical shape of several MNPs, which can be noticed in Figure 3. The polyhedral shape provides larger surface than sphere. Hence, the specific surface area of the ensemble of MNPs in the study is to some extent higher than that for the equivalent ensemble of exact spheres. The enlarged S_sp_ resulted in lower estimated values of d_s_ than that obtained from PSD.

XRD patterns of iron oxide MNPs are given in Figure 3c. The crystalline structure of MNPs corresponded to the inverse spinel lattice with a space group Fd3m. The lattice period was found, a = 0.8358 nm, which was larger than that for maghemite (γ-Fe_2_O_3_, a = 0.8346 nm) but lower than that for magnetite (Fe_3_O_4_, a = 0.8396) [36]. Based on the dependence between the lattice period of the spinel cell and the effective state of oxidation of Fe, the non-stoichiometric composition of MNPs was found Fe_2.75_O_4_.

Ferrofluids with MNPs were stable to aggregation and sedimentation. The average hydrodynamic diameter of MNPs in suspension measured by the dynamic light scattering was 56 nm, and it did not change during a month of observation. This value corresponds to the fifth mode of PSD and is therefore larger that the value of d_s_ given above, which stands for the second mode. The discrepancy between them stems from the polydispersity of the ensemble of MNPs. The value of hydrodynamic diameter correlates well with such data obtained for LTE iron oxide MNPs earlier [28] and corresponds to the individual particles in ferrofluid. The stability of ferrofluids was provided by the adsorption of negatively charged citrate anions on the surface of MNPs [28,29]. It gave negative net charge to the dispersed MNPs, provided their repulsion in the suspension, and prevented aggregation. The value of zeta-potential of the suspension was −40 mV, which was higher (in absolute value) than the threshold for the electrostatic stability of water-based suspensions [37].

No visible aggregation was observed in ferrofluid in the synthesis of ferrogels, which remained clear and non-opalescent after polymerization was completed. Thus, we assumed that individual MNPs present in ferrofluid remained in the ferrogel.

The main structural feature of ferrogel is the mesh size of its network. This can be estimated based on the equilibrium swelling ratio of a gel, which is the uptake of water by the dry polymeric network. The swelling ratio for all ferrogels is given in Table 1. There is a noticeable diminishing trend in values of apparent swelling ratio α, which was calculated according to Equation (1). Meanwhile, one should take into account that values of α included the weight of embedded MNPs. It is more correct to use the value of the swelling ratio for the PAAm network, α’ for the mesh size characterization. These values only slightly depended on the presence of embedded MNPs (see Table 1). We used the average value of α’ to characterize the mesh size of FG-I and FG-II series. It was 13.5 ± 0.8 for the FG-I series and 7.0 ± 0.4 for the FG-II series. The obtained values were then corrected, taking into account that dry residues contained both polymer and iron oxide MNPs. The degree of swelling related solely to the polyacrylamide network in ferrogels was independent of MNPs content in the gel. That is, iron oxide MNPs do not provide extra cross-linking of the network due to the adsorption of the sub-chains on their surface. 

Based on the equilibrium degree of swelling of the polymeric network (*α*’) the average number of monomer units in linear sub-chains between cross-links (*N_C_*) was evaluated using the Flory-Rehner equation [38]:

(7) $$N_{C}=\frac{V_{1}(0.5\alpha^{-1}-\alpha^{-1/3})}{V_{2}(\ln(1-\alpha^{-1})+\alpha^{-1}+\chi \alpha^{-2})}$$

where *V*_1_, *V*_2_ are molar volumes of a solvent and of a polymer respectively, *χ* is Flory-Huggins parameter for a polymer-solvent mixture. We used *V*_1_ = 18 cm^3^/mol (water), *V*_2_ = 56.2 cm^3^/mol (PAAm) and *χ* = 0.12. The last two values were obtained by means of quantum mechanics molecular modeling software package CAChe7.5. Equation (7) gave the number of monomer units in linear sub-chains *N_C_* = 55 for the PAAm network in the FG-I series and *N_C_* = 16 for the FG-II series. 

The equilibrium conformation of electrically neutral PAAm subchain in water is a random Gaussian coil with hindered rotation. The mean square end-to-end distance <R^2^>, which corresponds to the distance between adjacent cross-links (in other words the mesh size of the network) can be calculated according to the equation [39]:

(8) $$\langle R^{2}\rangle =Na^{2}\frac{1-\cos\theta }{1+\cos\theta }$$

where *N* is the number of bonds in the polymeric chain, *a* is the bond length, *θ* is the bond angle. We took *a* = 0.154 nm for the ordinary C–C bond, *θ* = 109.5° for the bond angle, and *N = 2N_C_* for the number of bonds. The mesh size of the network, calculated using Equation (8) is 2.4 nm for the FG-I series and 1.2 nm for the FG-II series. Both values are by an order of magnitude smaller than the average diameter of iron oxide MNPs (24.4 nm), which means that the cross-links of the polymeric network are closer to each other than the particle diameter. As a result, the MNPs cannot freely move inside the polymeric network of FG but are entrapped in it.

### 3.2. Deformation of Ferrogels and Their Electrical Properties

**Figure 4:**
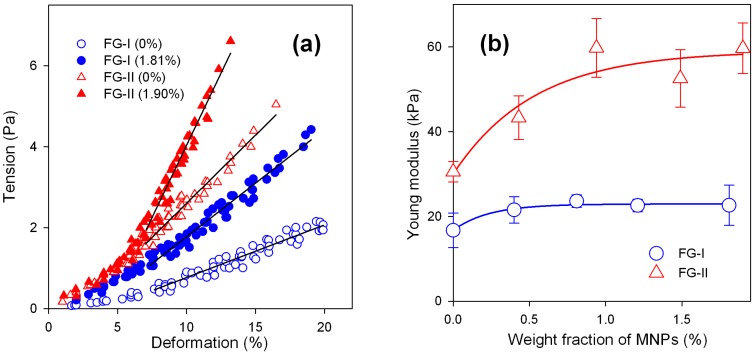
Typical deformation plots for ferrogels of FG-I and FG-II series. Open symbols correspond to unfilled (blank) gel, closed symbols correspond to ferrogel with the highest concentration of MNPs. Lines show the linear parts of the plots, which were used for the calculation of the Young modulus (**a**); Dependence of Young modulus of ferrogel on the weight fraction of MNPs. Lines are for an eye-guide only (**b**).

Figure 4a presents typical compression deformation plots for ferrogels of FG-I and FG-II series. Each plot is a combination of several step-wise deformation runs on the same sample. It is noticeable that deformation of soft ferrogels of the FG-I series takes place at lower values of tension than the deformation of ferrogels of the FG-II series (dense). This is a consequence of the smaller mesh size of the latter. In both series the embedding of MNPs in polymeric network substantially enlarges the tension, which is necessary to achieve a certain level of deformation. In this respect, embedded MNPs provide the enhancement of mechanical strength of a ferrogel equivalent to the additional cross-linking. Meanwhile, the swelling ratio of PAAm network (see Table 1) is not as sensitive to the presence of MNPs in the structure as mechanical strength. One common feature among plots given in Figure 4a is that there are two parts with a different slope in all of them. The initial part of the plot in the deformation range 0–7% is concave, while at higher deformations the plot is linear. Apparently, the concave part is the result of complex structural changes, which take place in the polymeric network under the applied force. The linear parts of the plots were taken for the calculation of Young modulus of ferrogels in both series.

**Figure 5:**
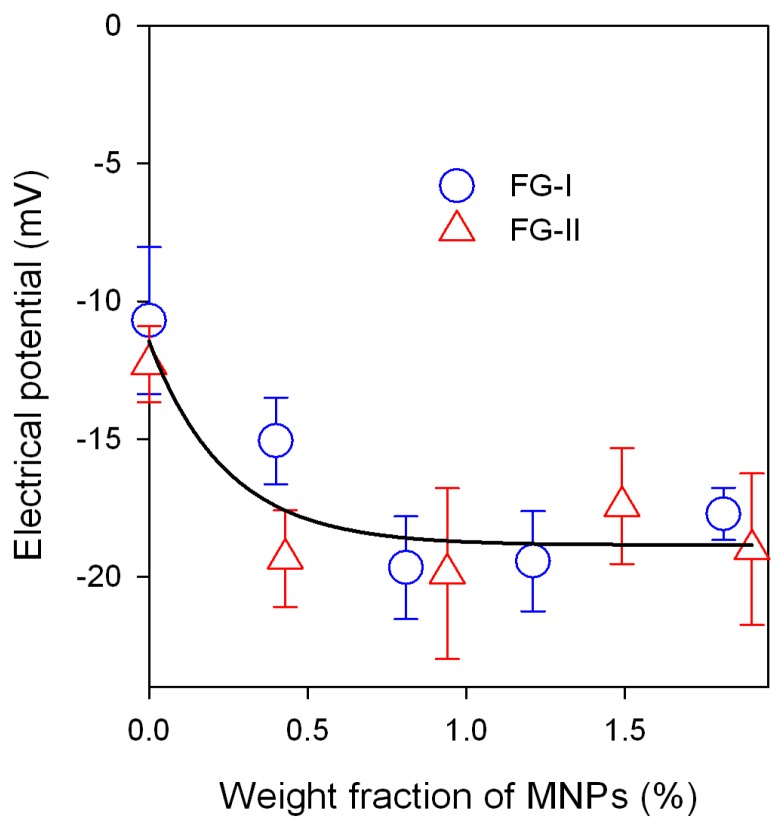
Dependence of the electrical potential of a ferrogel on the weight fraction of MNPs in FG-I and FG-II series. The line is for an eye-guide only.

Figure 4b shows the dependence of the Young modulus on the weight fraction of MNPs in ferrogel for FG-I and FG-II series. In FG-II (dense) series the embedding of MNPs in the polymeric network, even in a minimal concentration, resulted in a substantial increase of the Young modulus. In contrast, the addition of MNPs in a concentration larger than 1 wt % does not result in further increase of the modulus. In the FG-I (soft) series the influence of MNPs on the Young modulus of ferrogels is not as pronounced as in the FG-II series. Meanwhile, a limited increase in modulus with the weight fraction of MNPs can still be observed. There are two conclusions, which can be made from the data given in Figure 4. The first is that iron oxide MNPs in low concentration provide the increase in the Young modulus of ferrogels. The second is that this effect is enhanced by the density of the polymeric network. It is almost negligible if the mesh size of the network is large, and it becomes substantial if it decreases. This could also be attributed to the water uptake (swelling ratio) of the polymeric network. In other words, the influence of MNPs on the Young modulus becomes larger at a lower water uptake of the network. Figure 5 shows the dependence of the electrical potential of ferrogel on the weight fraction of iron oxide MNPs.

The potential in PAAm gel is negative and its value is ca. −10 mV. This value is low when compared to the typical values of the electrical potential for polyelectrolyte hydrogels such as gels of polyacrylic or polymethacrylic acid [40,41], which are larger by an order of magnitude. Certainly, it is because PAAm does not contain ionized groups in its polymeric sub-chains. In general, the electrical potential of the gel is Donnan potential [42], which is the result of the restricted ionic equilibrium on the gel boundary with supernatant. If there are ionic species, which cannot freely cross the gel boundary, it causes non-uniform distribution of free ions and eventually results in the existence of the electrical potential step on the boundary. In the case of polyelectrolyte gels, such unmovable ions are those attached to the polymeric sub-chains. 

There is a question as to why PAAm still show a low negative electrical potential even though they are non-ionic. For instance, it might be the result of a very small fraction of carboxylic residue in the PAAm structure due to the hydrolysis of AAm monomeric units.

The embedding of iron oxide MNPs in gel structure results in the increase of the negative values of the potential. Most likely it is due to the net negative electrical charge of MNPs themselves. As it was noted above, the ferrofluid taken for the synthesis of ferrogels was electrostatically stabilized by sodium citrate to prevent aggregation of MNPs. The zeta potential of the ferrofluid was −40 mV. This potential is located at the interface between the dense and the diffuse part of the double electrical layer at the surface of the MNPs. If MNPs are embedded into the PAAm network, they take the double electrical layer with them. As it was shown above, the mesh size of the network is by an order of magnitude smaller than the average diameter of MNPs. Thus, negatively charged MNPs cannot move across the ferrogel boundary and such a restriction gives rise to the native Donnan potential of the ferrogel.

It is noticeable in Figure 5 that the negative values of the electrical potential substantially increase at low concentration of MNPs and stay almost constant at higher concentration. There is no difference between the FG-I and FG-II series with respect to the electrical potential. It is the same for both series within the experimental error.

### 3.3. Magnetic Characterization of Nanoparticles and Ferrogels 

**Figure 6:**
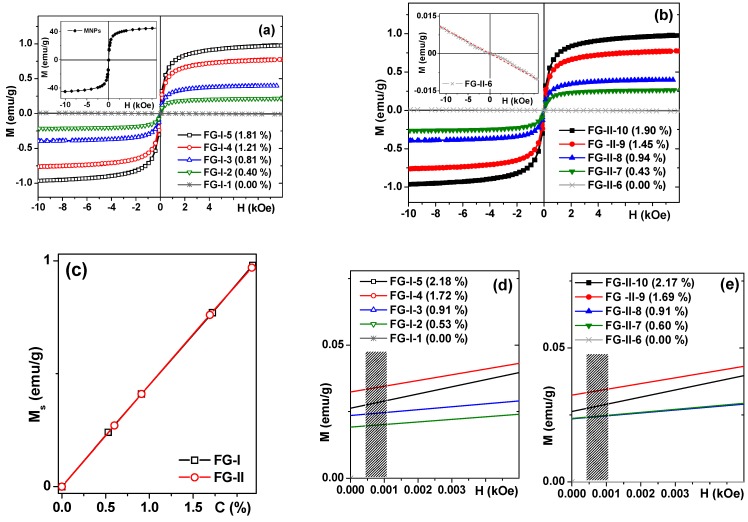
M(H) hysteresis loops of soft (**a**) and of dense ferrogels (**b**): in both cases concentrations of magnetic filler are obtained from the synthesis data. Inset for (**a**) shows the hysteresis loop of iron oxide MNPs from dried ferrofluid. Inset for (**b**) shows the hysteresis loop of pure gel. Concentration dependence of the saturation magnetization for soft and dense gels with different amount of magnetic filler (**c**). Parts of M(H) hysteresis loops of soft (**d**) and of dense ferrogels (**e**) approaching remanence: in both cases concentrations of magnetic filler are given for magnetic measurements data. Vertical bars indicate the field interval of 4 to 11 Oe corresponding to typical anisotropy fields of thin magnetic films used in sensor applications.

Figure 6 and Table 2 summarize the results of the magnetic measurements: M(H) hysteresis loops of soft ferrogels are given in Figure 6a and of dense ferrogels in Figure 6b. Although full saturation was not reached in the field of 10 kOe for the samples with high concentration of MNPs, we used the magnetization value for this field as the saturation magnetization M_s_ just for simplicity. The validity of such approximation was confirmed by the fact that M(H = 10 kOe) ≈ 0.95 × M(H = 70 kOe) [28]. For practical reasons of magnetic biodetection, magnetic field H = 1 kOe was sufficient. In all cases under consideration, including measurements of the MNPs dried from ferrofluid, very small coercivity of 8 Oe was observed at room temperature.

This can be explained by the presence of very small amounts of large particles due to the existence of particle size distribution (see Figure 3b). Even so, the behavior is quite close to the one expected for a superparamagnetic ensemble of iron oxide MNPs of this size. It is also important to mention that the contribution of pure gels was diamagnetic and very small in comparison with the magnetic signals of ferrogels. We have subtracted gel contributions when necessary from the ferrogel signals in order to obtain magnetic signals corresponding to the MNPs. 

Inset Figure 6a shows the hysteresis loop of MNPs with saturation magnetization about 45 emu/g, which corresponds quite well to the average size, d_s_ = 24.4 nm of MNPs [28,43]. The measurements of MNPs allow us to check the iron oxide concentration in ferrogels. Table 2 shows iron oxide concentrations obtained from the experimental data related to synthesis. Although these values are reliable, since the mass fraction of particles changes when manipulating the gel due to the inevitable loss of moisture, it is very difficult to estimate the MNPs concentrations from the synthesis data with high precision. Instead one can take advantage of magnetic measurements. Knowing the magnetization of the ferrogel in H = 10 kOe, we can calculate the concentration of the magnetic filler taking into account the magnetization of MNPs in the same magnetic field. The concentrations of MNPs obtained from M_s_ data were reasonably close to those calculated from the synthesis data (Table 2).

Figure 6c shows the concentration dependences of the saturation magnetization for soft and dense gels with different amounts of magnetic filler—a very good linear fit is evident in both cases under consideration. Figure 6d,e show parts of M(H) hysteresis loops of soft and dense ferrogels approaching remanence: in both cases concentrations of magnetic filler were recalculated for magnetic measurement data. Vertical bars indicate the field interval of 4–11 Oe, which correspond to typical anisotropy fields of thin magnetic films used in sensor applications. Here it becomes very clear that for efficient detection the magnetic moment must be of the order of 0.025 emu/g. For a ferrogel sample with a volume of about 18 mm^3^ (9 mm × 2 mm × 1 mm geometry), this means a magnetic moment of 0.0005 emu in the field near the anisotropy field. 

### 3.4. Characterization of the Multilayered MI Element

**Figure 7:**
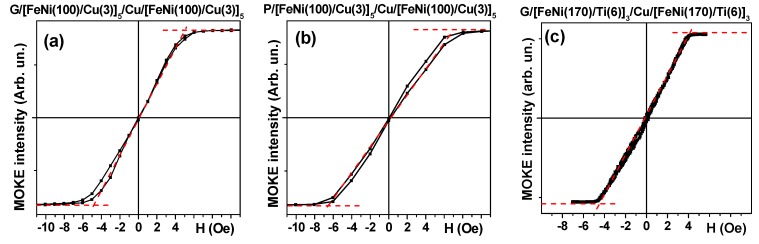
M(H) hysteresis loops of MI multilayered structures in the shape of rectangular elements. Numbers indicate thicknesses of the layers in nanometers. Thickness of the central Cu lead was equal to 500 nm in all cases under consideration. MI multilayers in the cases (**a**,**c**) were deposited onto glass substrates and onto cycloolefin COC flexible substrate in case (**b**). All units for the thickness of the layers are nanometers. The dashed red lines determine the anisotropy fields.

Figure 7 shows hysteresis loops of magnetic multilayered structures in the shape of MI elongated stripes. M(H) loops were measured by MOKE in the external magnetic field applied in the plane of the multilayered structure and parallel to the long side of the stripe, i.e., in the hard magnetization direction. The shape of the M(H) curves confirms that the application of an external magnetic field during the deposition of the multilayered structures did indeed result in the formation of the induced magnetic anisotropy with easy magnetization axes oriented in the plane of the film parallel to the short side of the sensitive element. The anisotropy fields for FeNi/Cu-based multilayers were about 7 Oe.

It is important to mention that both FeNi/Cu- based structures have quite similar magnetostatic characteristics. Previously, we conducted special studies to define the optimum parameters for FeNi/Ti-based multilayers deposition onto glass and flexible COC substrates [44]. The comparison of the M(H) loops (Figure 7) shows that FeNi/Cu- based structures with well-defined uniaxial induced magnetic anisotropy can be also successfully deposited onto both kinds of substrates. Although present day deposition techniques allow deposition of very sophisticated multilayered structures combining magnetic, conducting and insulating layers, simple structures with a smaller number of different types of layers always have a technological advantage. Actually, the deposition of [FeNi(170 nm)/Ti(6 nm)]_3_/Cu(500 nm)/[Ti(6 nm)/FeNi(170 nm)]_3_ multilayers requires the utilization of three targets, whereas the deposition of [FeNi(100 nm)/Cu(3 nm)]_3_/Cu(500 nm)/[Cu(3 nm)/FeNi(100 nm)]_5_ multilayers requires two targets during the sputtering process.

### 3.5. Model for Distribution of Electromagnetic Fields in Multilayered Film with Ferrogel

Let us consider a film structure [F/X]_n_/F/C/[F/X]_n_/F having the length *l* and width *w* < *l*. The film structure consists of a highly conductive central layer C of thickness 2*d*_0_ and two external magnetic multilayers consisting of soft magnetic layers F of thickness *d*_2_ and non-magnetic separating layers X of thickness *d*_1_. The total thickness 2*t* of the film structure is given by the following expression: 2*t* = 2*d*_0_ + 2*nd*_1_ + 2(*n* + 1)*d*_2_. It is assumed further that the material of the central layer and separating layers is the same, although the model can be extended to the case of different materials C and X. The layer of ferrogel with a thickness of *d*_3_ is placed onto the top surface of the film structure.

The driving electric field *e* = *e*_0_exp(−*i*ω*t*) is applied to the film structure, and the external magnetic field *H_e_* is parallel to the long side of the film. It is assumed that the film length and width are much higher than its thickness. Neglecting the edge effects, we consider that the electromagnetic fields depend only on the coordinate perpendicular to the film plane (*z*-coordinate).

In this approximation, the solution of Maxwell equations for the amplitudes of the longitudinal electric field and transverse magnetic field in the central and separating non-magnetic layers can be expressed as,

(9) $$\begin{array}{l} e_{1}^{\left(j\right)}=(c\lambda_{1}/4\pi \sigma_{1})[A_{1}^{\left(j\right)}\cosh(\lambda_{1}z)+B_{1}^{\left(j\right)}\sinh(\lambda_{1}z)]\;,\\ h_{1}^{\left(j\right)}=A_{1}^{\left(j\right)}\sinh(\lambda_{1}z)+B_{1}^{\left(j\right)}\cosh(\lambda_{1}z)\;. \end{array}$$

Here $e_{1}^{\left(j\right)}$ and $h_{1}^{\left(j\right)}$ are the amplitudes of the electric and magnetic field, *j* = 1,…, 2*n* + 1 is the non-magnetic layer number, $A_{1}^{\left(j\right)}$ and $B_{1}^{\left(j\right)}$ are the constants, *λ*_1_ = (1 − *i*)/*δ*_1_, *δ*_1_ = *c*/(2*πωσ*_1_)^1/2^ and *σ*_1_ are the skin depth and conductivity of the non-magnetic layers, respectively, and *c* is the velocity of light. Note that the expressions for the field amplitudes in the central layer have an asymmetric form with respect to the central plane of the structure, *z* = 0, due to the presence of ferrogel.

The field amplitudes $e_{2}^{\left(k\right)}$ and $h_{2}^{\left(k\right)}$ in the ferromagnetic layers are given by the following expressions:

(10) $$\begin{array}{l} e_{2}^{\left(k\right)}=(c\lambda_{2}/4\pi \sigma_{2})[A_{2}^{\left(k\right)}\cosh(\lambda_{2}z)+B_{2}^{\left(k\right)}\sinh(\lambda_{2}z)]\;,\\ h_{2}^{\left(k\right)}=A_{2}^{\left(k\right)}\sinh(\lambda_{2}z)+B_{2}^{\left(k\right)}\cosh(\lambda_{2}z)\;. \end{array}$$

Here *k* = 1,…, 2*n* + 2 is the ferromagnetic layer number, $A_{2}^{\left(k\right)}$ and $B_{2}^{\left(k\right)}$ are the constants, *λ*_2_ = (1 − *i*)/*δ*_2_, *δ*_2_ = *c*/(2π*ωμσ*_2_)^1/2^, *σ*_2_ and *μ* are the skin depth, conductivity and transverse permeability of the soft magnetic layers, respectively.

The solution of Maxwell equations in the ferrogel can be presented in the form:

(11) $$\begin{array}{l} e_{3}=[A_{3}\cosh(\lambda_{3}z)+B_{3}\sinh(\lambda_{3}z)]/\varepsilon^{1/2}\;,\\ h_{3}=A_{3}\sinh(\lambda_{3}z)+B_{3}\cosh(\lambda_{3}z)\;. \end{array}$$

Here *A*_3_ and *B*_3_ are the constants, *λ*_3_ = −*iωε*^1/2^/*c* and *ε* is the permittivity of ferrogel layer.

For the external regions, *z* < −*t* and *z* > *t* + *d*_3_, the approximate solution for the fields can be written as [44,45,46],

(12) $$\begin{array}{l} e_{m}=C_{m}\frac{i\omega }{c}\left[\frac{l}{2w}\log\left(\frac{R+w}{R-w}\right)-\frac{2z}{w}\arctan\left(\frac{wl}{2Rz}\right)+\frac{1}{2}\log\left(\frac{R+l}{R-l}\right)\right]\;,\\ h_{m}=-C_{m}\frac{4lz}{R}\left[\frac{R^{2}+4z^{2}}{4R^{2}z^{2}+l^{2}w^{2}}-\frac{1}{R^{2}-w^{2}}-\frac{1}{R^{2}-l^{2}}\right]+C_{m}\frac{2}{w}\arctan\left(\frac{wl}{2Rz}\right)\;. \end{array}$$

Here *m* = 4 and *m* = 5, correspond to the bottom and top external region, respectively, *C_m_* are the constants and *R* = (*l* ^2^+ *w*^2^ + 4*z*^2^)^1/2^. Note that expression (11) was obtained assuming that *w* << *l* and allows one to describe the distribution of the electromagnetic fields outside the film structure with ferrogel.

The constants in Equations (9)–(12) can be found from the continuity conditions for the amplitudes of the electric and magnetic fields at the interfaces between the ferromagnetic and non-magnetic layers. Furthermore, boundary conditions at the film structure surface and at the interface between the ferrogel and external region should be added.

Taking into account that the driving electric field is applied to the film structure only, the boundary conditions at the bottom surface of the film, *z* = −*t*, can be written in the following form:

(13) $$e_{2}^{\left(1\right)}(-t)=e_{4}(-t)+e_{0}\;,\;h_{2}^{\left(1\right)}(-t)=h_{4}(-t)\;.$$

Similar expressions can be found at the interface between the film and ferrogel, *z* = *t*,

(14) $$e_{2}^{\left(2n+2\right)}(t)=e_{3}(t)+e_{0}\;,\;h_{2}^{\left(2n+2\right)}(t)=h_{3}(t)\;.$$

The boundary conditions at the top surface of the ferrogel layer have the form:

(15) $$e_{3}(t+d_{3})=e_{5}(t+d_{3})\;,\;h_{3}(t+d_{3})=h_{5}(t+d_{3})\;.$$

The boundary conditions allow one to find the constants $A_{1}^{\left(j\right)}$, $B_{1}^{\left(j\right)}$, $A_{2}^{\left(k\right)}$, $B_{2}^{\left(k\right)}$, *A*_3_, *B*_3_, *C*_4_ and *C*_5_ in Equations (9)–(12) and describe completely the distribution of the electromagnetic fields. When the field distribution is obtained, the impedance *Z* of the film with the ferrogel layer can be found as a ratio of the applied potential difference *le*_0_ to the total current *I* flowing through the film:

(16) $$Z=\frac{le_{0}}{I}=\frac{le_{0}}{w\int_{-t}^{t}\sigma (z)e(z)dz}=\frac{2\pi l}{cw}\times \frac{e_{0}}{h_{2}^{\left(2n+2\right)}(t)-h_{2}^{\left(1\right)}(-t)}\;.$$

### 3.6. Effect of Ferrogel Layer on Film Permeability

The MI response of the multilayered film is controlled by the transverse permeability in the ferromagnetic layers. The transverse permeability depends on many factors, such as the domain structure, anisotropy axes distribution, mode of the magnetization variation, and so on. We assume that the value of the permeability in the ferromagnetic layers is governed by the magnetization rotation only. This approximation is valid at sufficiently high frequencies, when the domain-wall motion is damped [47]. We also suppose that the ferromagnetic layers have in-plane uniaxial anisotropy, and the direction of the anisotropy axes is close to the transverse one.

The influence of ferrogel on the MI can be attributed to stray fields induced by MNPs. The stray fields change the magnetization distribution in the soft magnetic layers of the film structure, and correspondingly, affect the film permeability. In general, the spatial distribution of the stray fields from MNPs can only be found by numerical calculations.

To describe qualitatively the effect of stray fields on the MI response, let us consider the following approximate model. It is assumed that the stray fields generate an effective field *H_p_* in the film structure. We suppose that the effective field is uniform over the film structure thickness. Note that a smooth variation of the effective field over the film thickness can be introduced in the model, with maximal value being at the top surface of the film and minimum value being at the bottom surface. Taking into account such approximate spatial distribution of the effective field results in a more complicated solution, however, it does not significantly influence the dependence of the impedances on the field and frequency.

It is assumed further that the value of *H_p_* is proportional to the concentration of MNPs in the ferrogel. This approximation seems to be reasonable since the magnetostatic measurements demonstrate that the ferrogel saturation magnetization increases linearly with the concentration of nanoparticles [2,27]. Experimental studies have shown that the ferrogels have S-shaped hysteresis loops [2,27,48]. To simplify calculations, we present the hysteresis curve by the linear field dependence of the ferrogel magnetization at low external fields.

Thus, we suppose that the effective stray field *H_p_* acts on the ferromagnetic layers of the film. The field *H_p_* has the opposite direction with respect to the effective angle φ of the ferrogel magnetization. The dependence of the angle φ on the external field can be approximated as follows:

(17) $$\sin\varphi =(H_{e}-H_{c})/H_{1}\;,$$

where *H_c_* is the coercivity and *H*_1_ is the external field corresponding to the approach of the ferrogel saturation magnetization.

The magnetization distribution in the ferromagnetic layers can be found by minimizing the free energy. Taking into account the effective stray field *H_p_*, the minimization procedure results in the following equation for the equilibrium magnetization angle *θ*:

(18) $$H_{a}\sin(\theta -\psi )\cos(\theta -\psi )+H_{p}\sin(\theta -\varphi )-H_{e}\cos\theta =0\;.$$

Here *H_a_* is the anisotropy field in the ferromagnetic layers and ψ is the deviation angle of the anisotropy axis from the transverse direction.

The solution of the linearized Landau-Lifshitz equation leads to the following expression for the transverse permeability *μ* in the ferromagnetic layers:

(19) $$\mu =1+\frac{\gamma 4\pi M(\gamma 4\pi M+\omega_{1}-i\kappa \omega )\sin^{2}\theta }{(\gamma 4\pi M+\omega_{1}-i\kappa \omega )(\omega_{2}-i\kappa \omega )-\omega^{2}}\;,$$

where *M* is the saturation magnetization of the ferromagnetic layers, *γ* is the gyromagnetic constant, *κ* is the Gilbert damping parameter, and

(20) $$\begin{array}{l} \omega_{1}=\gamma [H_{a}\cos^{2}(\theta -\psi )-H_{p}\cos(\theta -\varphi )+H_{e}\sin\theta ]\;,\\ \omega_{2}=\gamma [H_{a}\cos\{2(\theta -\psi )\}-H_{p}\cos(\theta -\varphi )+H_{e}\sin\theta ]\; \end{array}$$

Thus, the MI response in the multilayered film with a ferrogel layer can be calculated as follows. The first step is the calculation of the equilibrium magnetization angle in the ferromagnetic layers by means of Equations (17) and (18). The second step is the determination of the transverse permeability in the ferromagnetic layers by using Equations (19) and (20). Then, the corresponding values of the skin depth in the layers can be calculated, and the distribution of the fields is determined by means of Equations (9)–(12), taking into account boundary conditions. When the field distribution is found, the impedance of the film with ferrogel can be obtained by means of Equation (16).

### 3.7. Model Results

According to the model proposed, the ferrogel layer makes two contributions to the MI response. The first contribution is related to the high permittivity of the ferrogel, and the second one results from the stray fields of MNPs. Let us consider, first, the dielectric contribution of the pure gel without MNPs. In this case, the stray fields from the gel are equal to zero.

**Figure 8:**
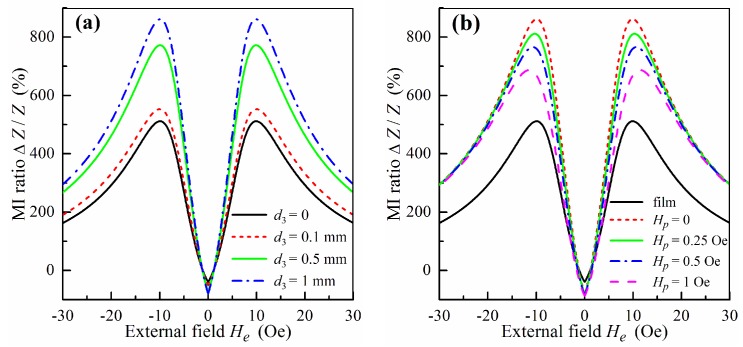
MI ratio Δ*Z*/*Z* as a function of the external field *H_e_* for gel at *f* = *ω*/2π = 100 MHz and different values of the ferrogel layer thickness *d*_3_ (**a**). MI ratio Δ*Z*/*Z* as a function of the external field *H_e_* for ferrogel at *f* = 100 MHz and different values of the effective stray field *H_p_*. The parameters of the ferrogel layer are *d*_3_ = 1 mm, *H_c_* = 4 Oe and *H*_1_ = 750 Oe (**b**). Parameters used for calculations are *l* = 1 cm, *w* = 0.01 cm, 2*d*_0_ = 500 nm, *d*_1_ = 5 nm, *d*_2_ = 100 nm, *n* = 4, *M* = 750 G, *H_a_* = 7.5 Oe, ψ= −0.05π, *σ*_1_ = 5 × 10^17^ s^−1^, *σ*_2_ = 4 × 10^16^ s^−1^, *κ* = 0.02 and *ε* = 70.

Figure 8a shows the calculated dependence of Δ*Z*/*Z* on the external field *H_e_* at the frequency of 100 MHz for different values of the gel layer thickness *d*_3_. The MI field dependence of the film structure without gel exhibits the typical two-peak behavior (curve 1 in Figure 8a). In the presence of the gel layer, the dependence has the same shape, however, the value of Δ*Z*/*Z* increases. This fact can be ascribed to the high permittivity of the gel, which influences the MI response of the film structure. It follows from Figure 8a that the MI ratio increases with the thickness of the gel layer. Note that a similar significant growth of the MI ratio in the presence of pure gel has been observed previously in experimental studies [2,48].

To describe the field dependence of the MI ratio for the film with the ferrogel layer we take the following values of coercivity *H_c_* and field *H*_1_ in Equation (17): *H_c_* = 4 Oe and *H*_1_ = 750 Oe. These values of the parameters correspond to the hysteresis loops measured in [2]. Figure 8b shows the field dependence of the MI ratio Δ*Z*/*Z* for the film without gel, film with pure gel and film with ferrogel at different values of the effective stray field *H_p_*.

**Figure 9:**
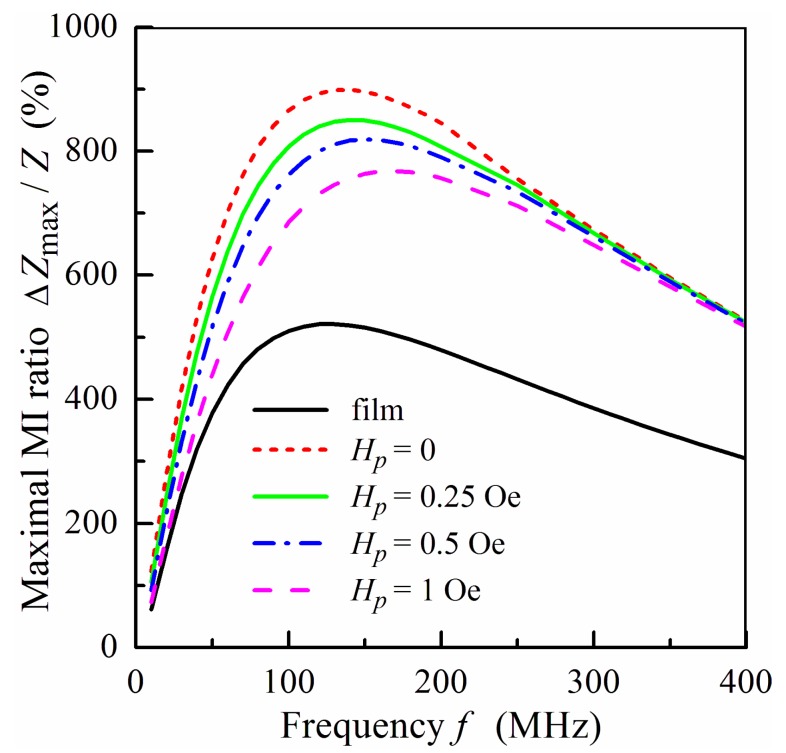
Frequency dependence of the maximum value of the MI ratio Δ*Z*_max_/*Z* for ferrogel at different values of the effective stray field *H_p_*. Other parameters used for calculations are the same as in Figure 8.

The frequency dependence of the maximum value of MI ratio Δ*Z*_max_/*Z* for the same samples is shown in Figure 9. An increase in the concentration of MNPs in the ferrogel leads to growth in the saturation magnetization of the ferrogel and to an enhancement of the effective stray field *H_p_*. As a result, the value of the MI ratio decreases with an increase in the concentration of MNPs in the ferrogel. The calculated dependences presented in Figure 8b and Figure 9 are in qualitative agreement with experimental results obtained in [2,48].

The model proposed allows one to explain qualitatively the main features of the experimental results concerning the MI response of multilayered films with a ferrogel layer [2,48]. It is demonstrated that high permittivity of the pure gel layer affects the MI response of the film structure, resulting in its significant enhancement. It is shown also that the contribution of the stray fields induced by MNPs in the ferrogel layer leads to a decrease in the MI ratio with an increase of the concentration of MNPs in the ferrogel. It should be noted that the model does not describe the essential shift of the maximum in the frequency dependence of Δ*Z*_max_/*Z* to lower frequencies with an increase in the concentration of MNPs in the ferrogel [2].

Note also, that the simplified presentation of the stray fields created by the MNPs by means of the effective field *H_p_* qualitatively describes the effect of the ferrogel layer on the MI of the multilayered film. 

However, this approximation has some restrictions. The first restriction is related to the fact that the effective stray field is assumed to be constant over the multilayered film thickness. The spatial variation of the effective stray field can be included in the model. However, this variation does not significantly affect the MI response. The second restriction is the introduction of the effective angle φ of the ferrogel magnetization to describe hysteresis loops of ferrogel layer at low external fields (see Equation (17)). A more general approach consists in the approximation of the experimental magnetization curves by analytical functions.

The main disadvantage of the model is as follows. It is evident that the effective stray field *H_p_* should be proportional to the concentration of the MNPs in the ferrogel. However, the coefficient of proportionality cannot be found in the framework of the approach proposed. To estimate the value of this coefficient, an approximate distribution of the stray fields should be found by means of a numerical solution for the magnetostatic equations.

### 3.8. Magnetoimpedance Measurements in Configuration of Biosensor Prototype 

Let us give some examples of MI responses of multilayered sensitive elements without and with ferrogels. In a previous section, comparative analysis of the MI behavior was based on our previous experimental research results and a new model approach was developed for ferrogel detection with a MI sensitive element showing good qualitative agreement with experimental data.

As mentioned before, in our previous works we used FeNi/Ti-based magnetic multilayers deposited onto rigid glass substrates and large gel pieces of 1.0 to 0.5 g weight having cylindrical or half cylinder shapes [2,35]. The measurements were strongly conditioned by time as the mass of the gel/ferrogels changed rapidly due to water loss. In the present work, we therefore developed a special protocol for the MI measurements with gels and FG taking into account the mass loss of the piece of gel or FG of the same shape and about 18 mg mass. 

**Figure 10:**
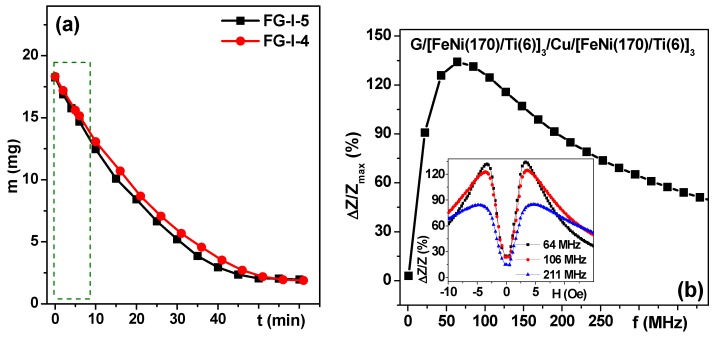
Mass loss for rectangular stripes of ferrogels used in MI measurements as a function of time drying in normal conditions (**a**); Frequency dependence of the maximum value of MI ratio Δ*Z*_max_/*Z* for multilayered structure deposited onto glass substrate. Inset shows field dependence of the Δ*Z*/*Z* ratio for characteristic selected frequencies. All units for the layers’ thickness are nanometers (**b**).

Figure 10a shows an example of the mass loss for rectangular stripes of ferrogels used in MI measurements. One can see that the dependence of the mass of the sample on the time t is non-linear for a time scale of about 1 h. In a traditional regime, the complete cycle of MI measurements from magnetic saturation in a positive magnetic field to saturation in a negative magnetic field and again from magnetic saturation in a negative magnetic field to magnetic saturation in a positive magnetic field (“down” and “up” branches, accordingly [49,50]) are usually made with an averaging procedure and take about 30–60 min depending on the number of measured frequencies. This means that for small pieces of gel a much faster measuring protocol must be elaborated.

There are two reasons for making an attempt to detect the pieces of ferrogels of a small size. The first, is the biomedical request to detect small tumors below 1 cm in size in order to insure early stage cancer diagnostics. The second, is a purely technical issue—modeling is only possible for thin layers of gel/ferrogel due to limitations in computer capacity. We therefore elaborated a special protocol for MI measurements in order to minimize the experimental errors and to ensure reproducible procedures with minimum measurement. Instead of complete measurements of “down” and “up” branches only the first half of the “down” branch was measured: the total time of the MI measurements in a decreasing field was limited to 3 min and characterized by the linear part for a total mass loss of 10% with respect to the initial mass (see Figure 10a). We were able to ensure repeatable way of measurements with very thin gels in order to proceed with modeling of the MI responses. The next step in this research process is to design a MI device with controlled humidity inside the measuring cell. 

**Figure 11:**
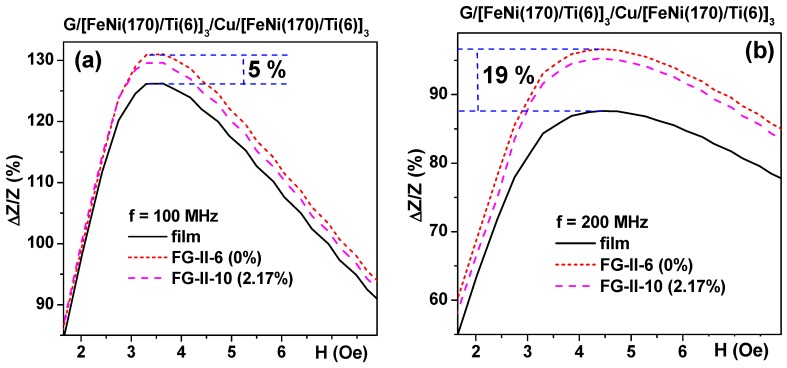
Field dependence of the Δ*Z*/*Z* ratio for thin film FeNi/Ti-based MI sensitive element measured either without gel/ferrogel or in the presence of gel or ferrogel with maximum available concentration of MNPs: (**a**) f = 100 MHz; (**b**) f = 200 MHz. Dashed lines show the difference between the MI element itself and the MI element covered by gel. G-glass substrate, numbers in the description of the multilayered structure are layer thicknesses. All units for the layers thicknesses are nanometers.

Figure 10b shows an example of a typical frequency dependence of a FeNi/Ti-based MI multilayered sensitive element. The inset shows the field dependence of the Δ*Z*/*Z* ratio for selected characteristic frequencies (near the maximum of Δ*Z*/*Z* ratio, and slightly and significantly above it). The two peak MI responses are very consistent with uniaxial magnetic anisotropy features evaluated from the shape of the hysteresis loops (Figure 7). Both frequency and field dependence of the observed types are widely described in the literature and understood in the framework of classic electrodynamics [18,20,51]. For the next step, the MI FeNi/Ti-based multilayered sensitive element was measured in the same conditions without, and with gel/ferrogel with maximum available concentration of MNPs (Figure 11a,b).

As before [2,35,49] the presence of the gel resulted in a sizable increase in the Δ*Z*/*Z* ratio near the anisotropy field of the MI element but the presence of a ferrogel of the same mass resulted in the displacement of the Δ*Z*/*Z* curve in the intermediate position between the position for the MI element itself and the MI element covered by pure gel. This is exactly what was predicted by the proposed electrodynamic model (compare Figure 8b and Figure 11a,b). It is also clear that the difference between the response of the MI element itself and the MI element covered by gel/ferrogel depends on the frequency of the driving current: in the case of G/[FeNi(170 nm)/Ti(6 nm)]_3_/Cu(500 nm)/[FeNi(170 nm)/Ti(6 nm)]_3_. Similar behavior was also observed for FeNi/Cu-based multilayered structures deposited onto flexible substrates for both kinds of gels (see also Table 2): in all cases the presence of a thin gel/ferrogel layer on the surface of the MI multilayered element resulted in an increase in the MI response near the anisotropy field. 

**Figure 12:**
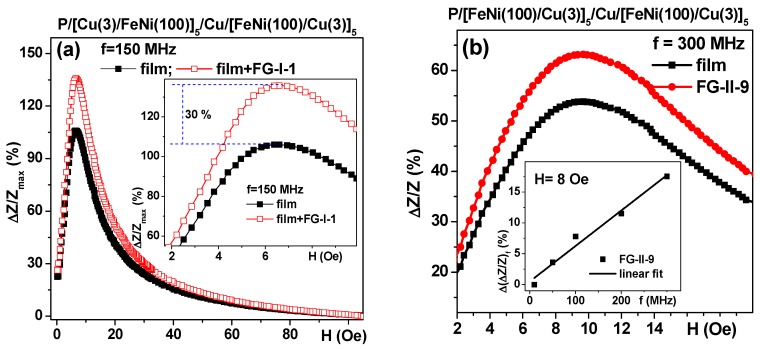
Field dependence of the Δ*Z*/*Z* ratio for thin film FeNi/Cu-based MI sensitive element measured either without gel/ferrogel or in the presence of gel (**a**) f = 150 MHz; (**b**) f = 300 MHz. Dashed lines (Inset (**a**)) show the difference between the MI element itself and the MI element covered by FG-I-1. P-polymer flexible substrate, numbers in the description of the multilayered structure are layer thicknesses. Inset (**b**) shows the difference in the field of 8 Oe between Δ*Z*/*Z* value for FG-II-9. All units for the layer thicknesses are nanometers.

In order to evaluate the difference between the Δ*Z*/*Z* value of the uncovered and gel covered MI element according to the type of frequency, we measured it for different frequencies (Figure 12b, Inset). For a mathematical representation the following equation was used for each one of the fixed frequencies: Δ(Δ*Z*/*Z*)_f_ = 100% × (Δ*Z*/*Z*_film+FG_ − Δ*Z*/*Z*_film_)/Δ*Z*/*Z*_film_). One can see clearly that the Δ(Δ*Z*/*Z*)_f_ parameter increases with an increase in the driving current frequency.

**Figure 13:**
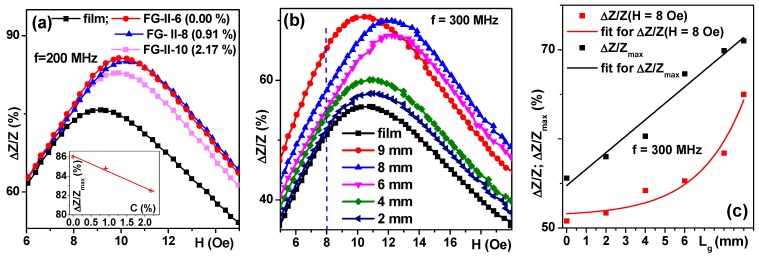
Field dependence of the Δ*Z*/*Z* ratio for thin film P/[FeNi(100)/Cu(3)]_5_/Cu(500 nm)/[FeNi(100)/Cu(3)]_5_. MI sensitive element measured either without gel/ferrogel or in the presence of gel and ferrogels (**a**) f = 200 MHz; (**b**,**c**) f = 300 MHz. Inset (**a**) shows the concentration dependence of Δ*Z*/*Z* ratio. Dashed line (**b**) shows the field of 8 Oe for which exponential dependence on the lengths of the gel sample was observed (**c**).

Despite the fact that evident qualitative agreement between the proposed model and the experimental data was obtained, for the practical purpose of biosensor application or simple use of MI for studies of gel materials, the experimental part of the measurements with gels required special efforts. Apart from an unusual time limitation due to gel dehydration, the measurements required fabricating gel samples of exactly the same shape and mass and exactly the same placement in the measuring system. For large samples previously studied in the MI-biosensor regime, both problems were less critical. Here, the major difficulties came not from the insufficient sensitivity of MI detector but rather from the difficulties of fabricating exactly the same samples and placing them into the system in the same way. Even so, we were able to obtain concentration dependences of the maximum of Δ*Z*/*Z* ratio for fixed frequencies (Figure 13) with a sensitivity of almost 2% per 1 wt % of MNPs. Here, we need to remember that all experiments were made with very diluted systems with very low concentrations of iron oxide MNPs. 

In order to show the extraordinary capacity of the MI element, we also measured the Δ*Z*/*Z* ratio for a fixed frequency of 300 MHz for the same ferrogel rectangular element which was cut step-by-step into shorter pieces of different length, L_g_ (Figure 13b). Although the procedure was not ideal (peaks for 8 and 6 mm were displaced toward higher fields), we were able to detect a tiny piece of about 4 mg of FG-II-10 ferrogel placed symmetrically in the centre of the MI element. This means the detection of about 0.09 mg of MNPs. Different parameters can be used for such detection. Figure 13c shows the comparison of Δ*Z*/*Z*(L_g_) and Δ*Z*/*Z*_max_(L_g_), where Δ*Z*/*Z*(L_g_) was taken for the external magnetic field of 8 Oe. Δ*Z*/*Z*(L_g_) shows exponential and Δ*Z*/*Z*_max_(L_g_) shows linear dependence on the gel stripe lengths.

There have been attempts to design a MI-based biosensor with different types of sensitive elements, such as amorphous ribbons, wires, and thin films [2,10,11,12,16,52,53]. They all have advantages and disadvantages, but the thin film direction seems to be the most promising. For multifunctional biosensor applications we expect the creation of a magnetic biosensor which would be capable of quantitatively monitoring living tissue functions, such as muscle contraction or evaluating a number of MNP loaded cells adhered onto the surface of a ferrogel implant near damaged tissue.

We were not able to design a biosensor prototype with controlled humidity inside the measuring chamber, but this is an urgent goal for the next study in order to avoid the limitations imposed by the short time for the measurements. Additional methodology should be elaborated to control the shape of small gel samples. Here, the biological technique of sample preparation might be useful [22]. Meanwhile, we have completed the next step in the development of a new generation of magnetic biosensors by the development of ferrogels incorporating magnetic nanoparticles into chemically crosslinked hydrogels mimicking some properties of the living systems and showing that the giant magnetoimpedance effect is promising for magnetic biosensor prototype development.

## 4. Conclusions and Outlook

In the present work, two series of ferrogels based on polyacrylamide (PAAm) with different chemical network densities (soft and dense) were synthesized by free-radical polymerization in aqueous solution with *N*,*N*’-methylene-diacrylamide as a cross-linker and iron oxide nanoparticles as a filler. These biocompatible maghemite Fe_2_O_3_ MNPs were fabricated by the electrophysical technique of laser target evaporation. Their mechanical (tension-deformation behavior, Young’s modulus), electrical (dependence of electrical potential of FG on the weight fraction of MNPs) and magnetic properties were comparatively analyzed. 

We developed a giant magnetoimpedance sensor prototype with multilayered FeNi-based sensitive elements deposited by magnetron sputtering onto glass or polymer substrates, which was adapted for FG studies. The MI measurements in the initial state and in the presence of FG with different concentrations of MNPs allowed the characterization of stray fields of the MNPs in the FG. This is the first report on ferrogel detection with a flexible MI sensitive element. This may open the possibility of creating prototypes for deformation evaluation. We proposed an electrodynamic model to describe the MI in multilayered film with a FG and found good qualitative agreement between the model and experimental data.

## Figures and Tables

**Table 1:** Composition and swelling ratio of ferrogels.

Number	FG Series	Concentration of MNPs, C (%)	Apparent Swelling Ratio, α (Unitless)	Swelling Ratio, α’ (Unitless)
1		0.00	14.4	14.4
2		0.40	13.0	13.7
3	FG-I	0.81	12.3	13.7
4		1.21	11.7	13.6
5		1.81	10.0	12.2
6		0.00	7.6	7.6
7		0.43	7.1	7.4
8	FG-II	0.94	6.4	6.8
9		1.45	6.0	6.6
10		1.90	6.0	6.7

**Table 2:** Magnetic properties of MNPs, gels and ferrogels: H_c_ is coercivity; M_s_ = M(H = 10 kOe).

Number	FG Series	Concentration of MNPs, % from Synthesis	H_c_, Oe	M_s_ for gel (emu/g)	Concentration of MNPs, % from M_s_ Data
1		0.00	0	~−0.01	0.00
2		0.40	13	0.24	0.53
3	FG-I	0.81	13	0.41	0.91
4		1.21	13	0.77	1.72
5		1.81	8	0.98	2.18
6		0.00	0	~−0.01	0.00
7		0.43	6	0.27	0.60
8	FG-II	0.94	6	0.41	0.91
9		1.45	7	0.76	1.69
10		1.90	7	0.97	2.17
11	MNPs	100	8	44.90	100.00
